# OTX2 Homeoprotein Functions in Adult Choroid Plexus

**DOI:** 10.3390/ijms22168951

**Published:** 2021-08-19

**Authors:** Anabelle Planques, Vanessa Oliveira Moreira, David Benacom, Clémence Bernard, Laurent Jourdren, Corinne Blugeon, Florent Dingli, Vanessa Masson, Damarys Loew, Alain Prochiantz, Ariel A. Di Nardo

**Affiliations:** 1 Centre for Interdisciplinary Research in Biology (CIRB), Collège de France, CNRS UMR7241, INSERM U1050, Labex MemoLife, PSL University, 75005 Paris, France; planques.anabelle@gmail.com (A.P.); vanessa.oliveira-moreira@college-de-france.fr (V.O.M.); david.benacom@college-de-france.fr (D.B.); clemence.bernard@kcl.ac.uk (C.B.); alain.prochiantz@college-de-france.fr (A.P.); 2 Genomics Core Facility, Institut de Biologie de l’ENS (IBENS), Département de Biologie, École Normale Supérieure, CNRS, INSERM, PSL University, 75005 Paris, France; jourdren@biologie.ens.fr (L.J.); blugeon@biologie.ens.fr (C.B.); 3 Laboratoire de Spectrométrie de Masse Protéomique, Centre de Recherche, Institut Curie, CEDEX 05, 75248 Paris, France; florent.dingli@curie.fr (F.D.); vanessa.masson@curie.fr (V.M.); damarys.loew@curie.fr (D.L.); 4 Institute of Neurosciences, Chinese Academy of Sciences, 320 Yue Yang Road, Shanghai 200031, China

**Keywords:** homeodomain, transcription factor, splicing, homeostasis

## Abstract

The choroid plexus is an important blood barrier that secretes cerebrospinal fluid, which essential for embryonic brain development and adult brain homeostasis. The OTX2 homeoprotein is a transcription factor that is critical for choroid plexus development and remains highly expressed in adult choroid plexus. Through RNA sequencing analyses of constitutive and conditional knockdown adult mouse models, we reveal putative functional roles for OTX2 in adult choroid plexus function, including cell signaling and adhesion, and show that OTX2 regulates the expression of factors that are secreted into the cerebrospinal fluid, notably transthyretin. We also show that *Otx2* expression impacts choroid plexus immune and stress responses, and affects splicing, leading to changes in the mRNA isoforms of proteins that are implicated in the oxidative stress response and DNA repair. Through mass spectrometry analysis of OTX2 protein partners in the choroid plexus, and in known non-cell-autonomous target regions, such as the visual cortex and subventricular zone, we identify putative targets that are involved in cell adhesion, chromatin structure, and RNA processing. Thus, OTX2 retains important roles for regulating choroid plexus function and brain homeostasis throughout life.

## 1. Introduction

The choroid plexus (ChP) epithelium is located in the brain ventricles and secretes cerebrospinal fluid (CSF) containing molecules that regulate embryonic brain development and adult brain homeostasis [1]. The ventricular system includes the two lateral ventricles (LVs) in each cerebral hemisphere, the central third ventricle of the forebrain diencephalon, and the central fourth ventricle (4V) in the hindbrain. This system is interconnected, allowing for CSF flow throughout, and is also connected via the 4V with the central canal of the spinal cord. The OTX2 homeoprotein transcription factor is critical for ChP embryonic development and functions [2]. Interestingly, temporal and spatial heterogeneity is evident, as the role of OTX2 evolves during development and differs between ChPs. For example, in late embryonic development, OTX2 is required for the maintenance of the 4V ChP, but not LV ChP [2]. Indeed, embryonic LV and 4V ChP show distinct gene expression patterns [3,4], suggesting different signaling properties. In the adult, OTX2 is still strongly expressed in the ChP [5], but its role has not been thoroughly investigated.

Homeoproteins are transcription factors that are important for embryonic development, and adult homeostasis and cell survival, and several homeoproteins have functions beyond transcription, including translation regulation, DNA repair, and signal transduction [6,7,8]. While several studies have explored the molecular partners and transcriptional targets of OTX2, they were typically restricted to embryonic contexts [9,10,11,12,13]. In the adult mouse, recent analyses of OTX2 protein and DNA targets have focused on the retina [14,15], visual cortex [16,17], and ventral tegmental area [18]. These studies revealed targets that are implicated in transcription, epigenetics, signal transduction, and homeostasis, and confirmed that OTX2 not only binds multiple sites across DNA, but also interacts with the machinery for RNA processing, export, and translation. To examine the role of OTX2 in adult ChP, we use a mouse model for constitutive heterozygous *Otx2* knockdown, and a model for the ChP-specific conditional knockdown of *Otx2*. Through transcriptomic analysis of LV and 4V ChPs, we reveal dysregulation of cell adhesion and membrane proteins, secreted factors, signaling factors, immune response, and oxidative stress response. Through mass spectrometry analysis of OTX2 partners in ChP and non-cell-autonomous target regions [19,20], including the ventricular–subventricular zone (SVZ), rostral migratory stream (RMS), and visual cortex (VCx), we identified putative targets that are involved in cell adhesion, chromatin structure, and RNA processing. We also performed splice variant analysis and confirmed, by acute viral shRNA-*Otx2* knockdown in the ChP of adult wildtype mice, that OTX2 can regulate the isoform distribution of genes involved in stress response and DNA repair. Taken together, our findings suggest that OTX2 has direct roles in ChP signaling, barrier, and surveillance functions.

## 2. Results and Discussion

### 2.1. Conditional and Constitutive Knockdown of Otx2 in Adult ChP

OTX2 is a key regulator of ChP and brain development, but its role in adult ChP is not well known. To gain an insight into its adult ChP functions, we performed RNA sequencing analysis with two mouse models. The first consisted of 3-month-old *Otx2^lox/lox^* mice for the conditional knockdown of *Otx2*, specifically in the ChP, through intracerebroventricular (icv) injections of Cre-Tat recombinant protein, which leads to a ~50% reduction in (mRNA) *Otx2* and a >70% reduction in OTX2 protein levels [5]. The ChPs from LV and 4V were dissected separately from both Cre-Tat-injected (Cre^+^*Otx2^lox/lox^*) and control vehicle-injected mice (Veh^+^*Otx2^lox/lox^*). While the bilateral stereotaxic injections of vehicle or Cre-Tat are performed only in the lateral ventricles, we have previously shown, with this protocol, that the level of *Otx2* knockdown in 4V ChP is proportional to that in LV ChP [19]. Indeed, we found a 48% decrease in (mRNA) *Otx2* (exon 2) in the LV ChP (vehicle, 7924 mean reads; Cre-Tat 4143 mean reads), and a 39% decrease in 4V ChP (vehicle, 6898 mean reads; Cre-Tat 4169 mean reads). The second model consisted of *Otx2^+/GFP^* mice as a constitutive heterozygous knockout mutant, with ~50% Otx2 protein levels compared to the wildtype [21]. For this model, given the significant overlap in gene expression changes in the LV and 4V of Cre^+^*Otx2^lox/lox^* mice (see below), only the 4V ChPs were dissected and pooled from 3-month-old wildtype and mutant mice. We found a 38% decrease in (mRNA) *Otx2* (exon 2) in 4V ChP (wildtype, 6399 mean reads; *Otx2^+/GFP^* 3957 mean reads).

**Figure 1:**
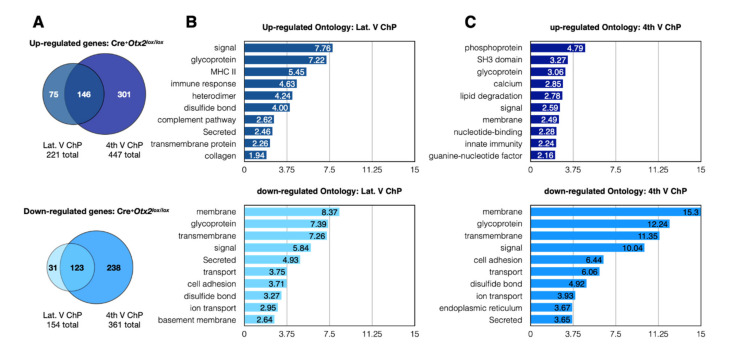
Changes in gene expression after conditional *Otx2* knockdown in choroid plexus. (**A**) Venn diagrams of the number of up- or down-regulated genes (*p-adj* < 0.05) from RNAseq analysis of ChP of Cre-Tat icv-injected *Otx2^lox/lox^* mice (Cre^+^*Otx2^lox/lox^*) compared to vehicle-injected mice. The diagrams compare the overlap between lateral ventricle (Lat. V ChP) and fourth ventricle (4th V ChP) differentially regulated genes; (**B**) Ontology analysis of differentially regulated genes in lateral ventricle ChP after conditional *Otx2* knockdown; (**C**) Ontology analysis of differentially regulated genes in fourth ventricle ChP after conditional *Otx2* knockdown.

The transcriptomics analysis of adult ChP showed highly expressed genes that are involved in energy metabolism, protein signaling, solute transport, cell adhesion, the cytoskeleton, and chaperone activity (Table 1). While not in the same order of gene expression level, this list compares favorably with those obtained from other ChP transcriptomics studies [4,22,23]. The conditional adult mouse knockdown of *Otx2* led to significant changes in the expression of 375 genes in LV ChP and 808 genes in 4V ChP (*p-adj* < 0.05). The top ten upregulated and downregulated genes show a range of functions, including solute transport, signaling, immune response, and trafficking (Table 2). While there is a significant overlap in the altered gene expression between the ChPs (Figure 1A), the 4V ChP seems to be more susceptible to loss of *Otx2* activity. The response to *Otx2* knockdown results in a rather even distribution of upregulation (522 genes) and downregulation (392 genes) when grouping both 4V and LV ChPs. However, ontological analysis reveals that the downregulated genes in both ChPs show higher levels of enrichment in significantly altered classes, suggesting that upregulated genes have more broadly distributed functions (Figure 1B,C). Interestingly, both ChPs have similar ontology enrichment in downregulated genes, indicating that *Otx2* is generally important for the expression of membrane proteins, glycoproteins, signaling proteins, and cell adhesion proteins. While some of these functions are recapitulated in the upregulated genes, there is much more heterogeneity between the LV and 4V ChP. The LV ChP shows more immune response ontology, while the 4V ChP shows more signaling-related ontology. This suggests that conditional knockdown of *Otx2* leads to altered ChP barrier function and ChP signaling, and can impact immune responses.

**Figure 2:**
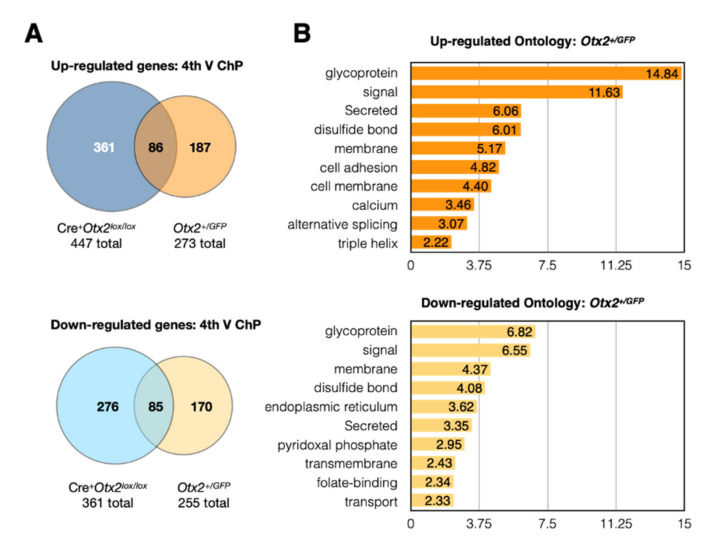
Gene expression in choroid plexus of *Otx2^+/GFP^* mice. (**A**) Venn diagrams of the number of up- or down-regulated genes (*p-adj* < 0.05) to compare overlap between fourth ventricle (4th V ChP) changes in *Otx2^+/GFP^* mice and in Cre-Tat icv-injected *Otx2^lox/lox^* mice (Cre^+^*Otx2^lox/lox^*); (**B**) Ontology analysis of differentially regulated genes in *Otx2^+/GFP^* mice.

The constitutive heterozygote *Otx2^+/GFP^* adult mice showed significant expression changes in 528 genes of the 4V ChP (*p-adj* < 0.05), which is comparatively less than for conditional *Otx2* knockdown in the 4V ChP (Figure 2A). Given that fewer genes are deregulated in this constitutive model, this suggests that compensatory mechanisms for countering reduced OTX2 levels were activated during development. The changes in gene expression were relatively balanced between upregulation (273 genes) and downregulation (255 genes), and ontology analysis revealed shared terms, including glycoprotein, signal, membrane-related, and secreted proteins (Figure 2B). The upregulated genes are also enriched for cell adhesion and alternative splicing, while downregulated genes are enriched for trafficking and transport. This suggests that brain-wide and life-long knockdown of *Otx2* leads to altered ChP signaling, barrier functions, and brain homeostasis.

We hypothesized that genes that were deregulated in both conditional and constitutive models could be either direct targets of OTX2 transcription regulation or targets of important OTX2-dependent pathways. Comparison of gene expression changes in 4V ChP of these two models revealed an overlap of more than 80 genes, in both the upregulated and downregulated repertoires (Figure 2A). This represented about half of the identified expression changes in *Otx2^+/GFP^* mice, but less than a third of the changes in the conditional model. When genes from LV ChP conditional *Otx2* knockdown are included in the analysis, we identified 42 genes that are globally upregulated and 34 genes that are globally downregulated (Table 3). To determine whether this list contains direct OTX2 transcription targets, we compared it with OTX2 chromatin immunoprecipitation experiments that were previously performed in mouse embryonic brain [9] or adult retina [14]. However, we found almost no overlap, with only *Ttr* being a common target. This suggests that the transcription-related activity of OTX2 is different in the adult choroid plexus and/or that these deregulated genes are downstream targets of OTX2-dependent pathways. It will be necessary to perform ChIPseq or CUT&RUN analysis of adult choroid plexus, to distinguish between these possibilities. Taken together, our analysis identifies new potential functions for *Otx2* in the adult brain. We found upregulation of immune factors, specifically in the conditional *Otx2* loss-of-function model, and deregulation of genes involved in cellular adhesion, trafficking, signaling, and secretion, in both knockdown models, suggesting altered ChP function and disruption of the ChP barriers.

### 2.2. Altered Expression of ChP Secreted Factors

Given that our various ontology analyses often evoked secreted factors, we focused on ChP factors secreted in CSF and implicated in embryonic and/or adult neurogenesis (Table 4), which is one of the identified functions of adult ChP [19,23,24]. The factors implicated in embryonic neurogenesis include SHH, BMPs, and WNTs [25]. While *Shh* expression was not observed (mean reads < 1) in either ChPs of the wildtype mice, which is consistent with published data [3], the 4V ChP of *Otx2^+/GFP^* mice (but not the ChPs of conditional *Otx2* ChP knockdown mice) showed a significant increase in *Shh* expression. Between the various *Bmp* and *Wnt* family genes, only *Bmp7* and *Wnt2b* were differentially expressed in Cre^+^*Otx2^lox/lox^* mice, as compared to Veh^+^*Otx2^lox/lox^* mice. Canonical Wnt signaling is perturbed in embryos with *Otx2* 4V ChP knockdown, which was attributed to the dysregulation of Wnt modulators, including *Rspo1*, *Sfrp2*, *Sostdc1*, *Tgm2*, and *Wnt4,* and to the increased levels of WNT4 and TGM2 in the CSF of mutant mice [2]. While *Rspo1*, *Sfrp2*, and *Wnt4* were very poorly expressed and unchanged in both the LV and 4V ChP of Cre^+^*Otx2^lox/lox^* adult mice, the expression of *Sfrp1*, *Sostdc1*, and *Tgm2* were significantly changed in 4V ChP (Table 4), suggesting adult OTX2 retains some embryonic functions. To further explore this hypothesis, we compared our RNA sequencing analysis of 4V ChP Cre^+^*Otx2^lox/lox^* to the previous microarray analysis of embryonic *Otx2* knockdown, specifically in the hindbrain ChP, performed by Götz and colleagues [2]. They found 340 significantly (FDR < 10%, >2-fold change) expressed genes, with 135 genes upregulated and 225 genes downregulated. Compared to our adult knockdown, there was less than a 20% overlap with upregulated genes (24 of 135 genes) and an almost 30% overlap with downregulated genes (62 of 225 genes). Some of these genes are found among the top ten dysregulated 4V genes (Table 2) and the globally altered genes (Table 3), and they have functions related to cell adhesion, trafficking, and secretion. Given that *Otx2* knockdown experiments in late embryonic development showed that OTX2 is necessary for 4V, but not LV, ChP maintenance [2], our results suggest that adult *Otx2* expression could retain this maintenance function in 4V ChP.

We have previously shown that OTX2 that is secreted into the CSF from the ChP, can regulate adult neurogenesis non-cell autonomously, by transferring into the astrocytes in the SVZ and RMS, thereby affecting neuroblast migration [19]. This study also showed that ChP *Otx2* knockdown in Cre^+^*Otx2^lox/lox^* adult mice, which will impact both cell- and non-cell-autonomous activity, also led to significantly reduced SVZ neurogenesis, suggesting that OTX2 may regulate secreted factors that are implicated in adult neurogenesis, through cell-autonomous effects in the ChP. IGF2 and SLIT1/2 regulate both embryonic and adult neurogenesis [24,26,27,28]. While *Slit1* is not expressed and *Slit3* is only weakly expressed in adult ChP, *Slit2* is highly expressed, but shows no significant change in expression in the ChP, with reduced *Otx2*. Admittedly, there is a trend towards increased *Slit2* expression and we cannot exclude the potential for biological relevance. *Igf2* was significantly downregulated, more than 2-fold, in all the ChPs, upon *Otx2* knockdown. However, there was a concomitant downregulation in *Igfbp2*, which can inhibit IGF2, suggesting that the level of IGF2 activity could be maintained through the compensatory reduction in inhibiting factors. Other factors influencing adult neurogenesis include amphiregulin (AREG) [29], FGF2 [30,31,32], and TGF-α [33], yet we found no significant change in their expression (and no detectible expression of *Areg*). Finally, other factors show more change in gene expression after acute *Otx2* knockdown compared to constitutive knockdown. TGF-β negatively regulates adult neurogenesis [34], and *Tgf-β2* is downregulated in both the ChPs of Cre^+^*Otx2^lox/lox^* mice (Table 4). Taken together, these minimal or compensatory changes in specific secreted signaling factors suggest that *Otx2* expression in the ChP could have only a minor cell-autonomous role in regulating adult neurogenesis. This hypothesis is consistent with similar levels of decrease in adult neurogenesis, observed with both this ChP *Otx2* knockdown model and the non-cell-autonomous-only OTX2 knockdown mouse model [19].

### 2.3. Altered Expression of Immune and Stress Factors

**Figure 3:**
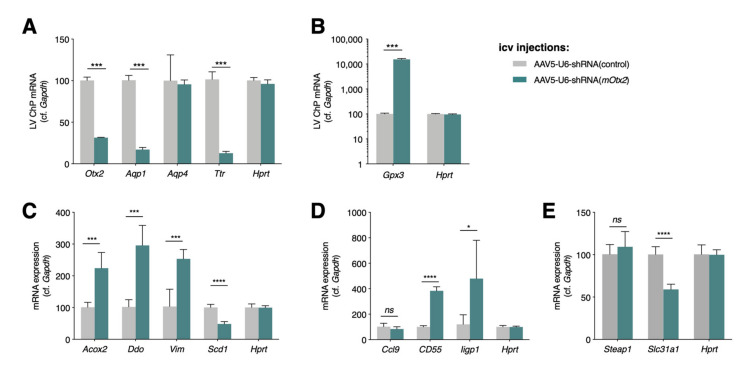
OTX2 regulates choroid plexus expression of oxidative stress, immune system, and metal transport genes. Quantitative PCR analysis of lateral ventricle (LV) ChP gene expression in wildtype mice after viral expression of shRNA against mouse (mRNA) *Otx2* (shRNA-*mOtx2*) compared to control shRNA. (**A**) Analysis of control genes to validate shRNA-*Otx2* activity; (**B**) *Otx2* knockdown induces high expression of *Gpx3*; (**C**) Analysis of select genes involved in oxidative stress response; (**D**) Analysis of select genes involved in immune system response; (**E**) Analysis of select genes involved in metal ion transport (all values: mean ± SEM; *n* = 5; *t*-test; * *p* < 0.05, *** *p* < 0.001, **** *p* < 0.0001).

Given the altered expression of homeostasis and stress response-related factors in both the ChPs of conditional *Otx2* knockdown mice, we turned to the viral expression of shRNA-*Otx2* in LV and 4V ChPs. Intracerebroventricular-injected AAV5 results in ChP-specific expression [35,36], and provides a tool to acutely affect Otx2 expression in any mouse model. We validated this model by qPCR analysis, which showed a 69% decrease in (mRNA) *Otx2* and a concomitant very large decrease in the expression of a known direct transcriptional target, transthyretin (*Ttr*) (Figure 3A). Comparing models, the decrease in (mRNA) *Ttr* in Cre^+^*Otx2^lox/lox^* mice was 45%, while it was 87% in the shRNA-*Otx2* mice, which suggests that viral knockdown provides a more robust effect. TTR, the most highly expressed protein in ChP (Table 1), is secreted into CSF and transports thyroxin and retinol-binding protein, and has a role in regulating cognition and memory, psychological health, and emotions (for a recent review, see [37]), suggesting that OTX2 levels can potentially impact similar brain functions. Furthermore, the downregulation of aquaporin 1 (*Aqp1)* (Table 3, Figure 3A) was also confirmed, with *Aqp4* as a negative control, suggesting that OTX2 can also regulate CSF water homeostasis.

In keeping with roles in brain homeostasis and surveillance, we also chose targets from ontology analysis (Figure 1), in functions related to oxidative stress, immune response, and metal ion transport. A surprising finding was the over 100-fold increase in glutathione peroxidase 3 (*Gpx3*), an extracellular enzyme that catalyzes the reduction in peroxidases and protects cells from oxidative damage, suggesting that a loss of OTX2 has a significant impact on cell physiology (Figure 3B). Other compensatory mechanisms against oxidative stress include decreased fatty acid oxidation (*Scd1*), increased peroxisome function (*Acox2*, *Ddo*), for countering oxidative stress and inflammation [38], and changes in the structural cell response (*Vim*) [39] (Figure 3C). Concerning the immune response (Figure 3D), we tested a complement activation factor (*CD55*), an inflammatory response chemokine (*Ccl9*), and an innate immune response factor (*Iigp1*). The direction of change in the expression of all of these factors, upon acute viral *Otx2* knockdown, was consistent with the constitutive and conditional mouse models. It remains unclear whether the loss of OTX2 provokes oxidative stress, and thus indirect activation of genes such as *Gpx3*, or whether OTX2 regulates the genes involved in reactive oxygen species signaling and/or stress response. Finally, given their role in brain homeostasis, we also quantified factors related to metal ion transport (*Steap1* and *Slc31a*) (Figure 3E), which had altered expression in the conditional Cre^+^*Otx2^lox/lox^* mice. Only *Slc31a*, which transports copper ion, had concomitant reduced expression upon *Otx2* knockdown. These findings suggest that ChP function is greatly impacted by *Otx2* expression level, opening the question of whether *Otx2* overexpression in ChP would also deregulate homeostasis and illicit immune responses in a wildtype context or, on the contrary, rescue deficits in homeostasis in an aged or diseased animal.

### 2.4. Otx2 Protein Interactions

To further analyze OTX2 function in adult ChP, we performed several OTX2 co-immunoprecipitation (co-IP) experiments with mass spectrometry (MS) analysis, to identify potential protein partners. We previously discovered that OTX2 protein is secreted by the ChP into CSF, and accumulates non-cell autonomously in SVZ and RMS astrocytes [19] and VCx parvalbumin cells [5,40]. The identification of alternate protein partners in cell-autonomous and non-cell-autonomous contexts would suggest that OTX2 takes on specific roles after transferring between cells. To test this hypothesis, and to reinforce ChP analysis, we also performed OTX2 co-IP on lysates from adult mouse SVZ, RMS, and VCx.

We used three criteria to obtain a list of potential OTX2 protein interactions in the four brain structures (Table 5), as follows: (i) unique proteins with three or more peptides identified exclusively in OTX2 compared to IgG co-IP samples (unique protein, ≥3 peptides); (ii) proteins identified with three or more peptides in OTX2 co-IP samples and having a relative peptide difference greater than 50% compared to IgG co-IP (selected protein, ≥50% rel. ∆); and (iii) all small proteins (≤25 kDa) exclusive to OTX2 co-IP samples, regardless of peptide number (unique small protein, ≤25 kDa), given that they have fewer identifiable MS peptides. These lists were used for comparison between structures and for ontology analysis. We generated a list of 60 high-confidence protein partners of OTX2 in ChP that were common to all three ChP samples (Table 6). These proteins cover several functions, including cell adhesion, cell trafficking, cell signaling, metabolism, RNA binding, RNA processing, transcription, chromatin structure, and DNA repair. Interestingly, more than 10% (eight proteins) belong to the “tier 1” proteins identified in stress granules [41], which are involved in translational control and post-transcriptional regulation. Although this functional class was not identified by KEGG pathway analysis (see below), this is likely due to the absence of annotation, given the only recent emergence of updated comprehensive inventories of stress granule proteins. Thus, we can only hypothesize that OTX2 interacts with these granules, although this putative function is given weight by the presence of the PAX1 homeoprotein among the “tier 1” proteins, by the in vivo interaction between the EMX2 homeoprotein with the translation initiation factor eIF4E [42], and by the involvement of the PROX1 homeoprotein in liquid–liquid phase separation [43], which also underlies stress granule assembly [41]. Also of note are the putative partners MECP2 and MOV10, given that OTX2 has been shown to regulate MECP2 foci in the postnatal mouse visual and auditory cortex [16], and that the EN1 homeoprotein is involved in LINE-1 regulation [44].

Few OTX2 partners have been biochemically and functionally validated. One key partner during embryogenesis is MEIS2, which is a co-activator for mesencephalon specification [45]. *Meis2* is expressed in ChP at low levels (170 mean reads in 4V ChP; 31 mean reads in LV ChP), as compared to OTX2 (5954 mean reads in 4V ChP and 6829 mean reads in LV ChP), and appears not to be a major partner of OTX2 in ChP. TLE4 is another identified protein partner of OTX2 during development, and allows repression of mesencephalon fate [46]. Despite its expression in ChP (798 mean reads in 4V ChP and 689 mean reads in LV ChP), TLE4 was not identified in our OTX2 co-IP, although this could be due to TLE4 being under the limit of detection or having peptides that are too hydrophobic or hydrophilic for MS detection. The potential absence of TLE4 suggests that OTX2 protein interactions depend strongly on the cell type and developmental context.

**Figure 4:**
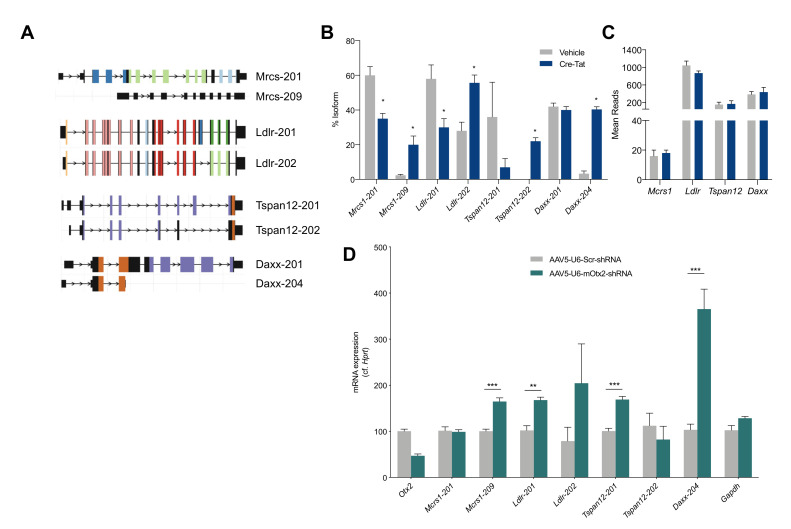
Analysis of splice variations induced by *Otx2* knockdown in choroid plexus. (**A**) Selected isoforms of genes of interest. Locus length is in arbitrary units. Colors represent different protein domains within a given gene; (**B**) Isoform usage, shown as % of total isoforms, in lateral ventricle (LV) ChP from vehicle and Cre-Tat icv-injected *Otx2^lox/lox^* mice; (**C**) Mean reads from transcriptomic analysis of LV ChP from vehicle and Cre-Tat icv-injected *Otx2^lox/lox^* mice; (**D**) Quantitative PCR analysis of LV ChP isoform expression in wildtype mice after viral expression of shRNA against mouse (mRNA) *Otx2* (shRNA-*Otx2*) (all values: mean ± SEM; *n* = 5; *t*-test; * *p* < 0.05, ** *p* < 0.01, *** *p* < 0.001).

To identify novel OTX2 protein partners that are ubiquitous throughout the brain, we compared the lists from the four brain structures, as follows: ChP (pooled LV and 4V), SVZ, RMS, and VCx. Few high-confidence proteins (selected protein ≥ 50% rel. ∆), 14 in total, were common to the three non-cell-autonomous structures (Table 7). Of these 14 common proteins, 5 were also identified in the ChP. Interestingly, these top-ranked proteins include FIG4, VAC14, and PIKFYVE, which play a role in phosphatidylinositol(3,5)bisphosphate [PI(3,5)P2] regulation, in multivesicular body (MVB) biogenesis, and in endosome autophagy and trafficking [47], suggesting that OTX2 plays a role in vesicle transport or is carried via MVBs. Given that MVBs can give rise to extracellular vesicles, interaction with OTX2 may reinforce its role in regulating the pathways of extracellular protein expression that are identified in our RNA sequencing analysis.

To identify potential differences between cell-autonomous and non-cell-autonomous partners, we performed KEGG pathway analysis on OTX2 protein partners, for all structures individually (Table 8). No dramatic differences were found between the structures, suggesting conserved roles of OTX2 in cell-autonomous and non-cell-autonomous OTX2 target structures. Common to nearly all structures are metabolic pathways, RNA transport, oxidative phosphorylation, RNA processing, and spliceosome. Pathways that are specific to the ChP pertain to the maintenance of tight junctions, protein processing, and actin cytoskeleton regulation. Downregulation of the cell adhesion class was also identified in the RNAseq analysis of conditional Cre^+^*Otx2-lox* mice (Figure 1), suggesting a direct involvement of OTX2 both in gene regulation and cellular functions for cell-autonomous ChP maintenance. Of the 14 proteins in common between VCx, SVZ, and RMS, eight of them are involved in RNA processing, suggesting a novel function for OTX2. Although the spliceosome pathway was also enriched in ChP, these proteins stand out for their involvement in the U5 snRNP complex, exon junction complex, or mRNA export complex, whereas the spliceosome proteins identified in the ChP are either splicing co-factors, part of the SMN complex, or part of the U2 snRNP complex. Interestingly, OTX2 has been shown to bind the initiation factor eIF4e in GST pull-down experiments [42], while other homeoproteins have been shown to bind translation machinery [6,42,48] that is implicated in RNA export, transport, and translation. Taking the high-confidence partners together with KEGG pathway analysis, cell-autonomous OTX2 is likely implicated in the regulation of genomic landscape, the regulation and processing of RNA, the trafficking of signals, and the maintenance of cellular adhesion, while non-cell-autonomous OTX2 is more implicated in the processing of RNA.

### 2.5. Splice Variant Analysis

Given the high confidence of OTX2 interaction with spliceosome pathway proteins, we extended the analysis of our transcriptomic data of LV ChP from Cre^+^*Otx2^lox/lox^* mice to measure changes in splice variants. The isoform usage was found to be significantly changed in the coding transcripts for only four genes (*Mcrs*, *Ldlr*, *Tspan12*, and *Daxx*), and generally for only two isoforms among the splice variants (Figure 4A,B). These genes showed no change in overall expression upon *Otx2* knockdown (Figure 4C). Through acute *Otx2* knockdown by the viral expression of shRNA-*Otx2*, we confirmed a significant increase in the expression of only the *Mcrs-209* and *Daxx-204* isoforms, as other isoforms either did not change significantly or changed in the opposite direction (Figure 4D). Interestingly, MCRS and DAXX interact within a protein complex with various nuclear functions, including transcription regulation, chromatin remodeling, and DNA repair. Further research is needed to determine the functional consequences of these changes in the distribution of transcript isoforms.

Homeoproteins have been postulated to regulate transcript splicing. The PAX6 homeoprotein has been shown to modulate splicing machinery, such that changes in *Pax6* expression alter the population of tenascin-C splice variants without changing the total tenascin-C expression [49], while the CDX2 homeoprotein interacts with splicing machinery [50]. Regarding OTX2, its protein interactome in the adult retina revealed putative RNA processing partners, such as SFPQ and U2AF [15], while in the ChP and non-cell-autonomous structures, we found the potential partners ACIN1, DDX41, DDX46, HNRPLL, and PRRC2A, RBM25, RBM39, SF3A1, SF3B1, SNRNP200, and SRRM2 (Table 6 and Table 7). It remains to be determined whether OTX2 controls ChP splicing activity through direct interaction with splicing factors and/or by regulating their expression.

## 3. Materials and Methods

### 3.1. Animal Ethics Statement

All animal procedures, including housing, were carried out in accordance with the recommendations of the European Economic Community (2010/63/UE) and the French National Committee (2013/118). For surgical procedures, animals were anesthetized with xylazine (Rompun 2%, 5 mg/kg) and ketamine (Imalgene 500, 80 mg/kg). For biochemical analysis, mice either underwent transcardial perfusion or were sacrificed by cervical elongation. 

### 3.2. Animals and Stereotaxic Surgery

*Otx2^lox/lox^* mice were kindly donated by T. Lamonerie [51] and *Otx2^+/GFP^* mice by A. Simeone [52]. Three-month-old *Otx2^lox/lox^* mice were injected with Cre-Tat or vehicle as described in [19] and housed for 15 days after surgery. The following serotype 5 adeno-associated viruses (AAV5) were purchased from Vector Biolabs (Malvern, PA, USA): AAV5-CMV-EGFP-U6-shRNA (control); and AAV5-CMV-EGFP-U6-shRNA (mOtx2). High-titer AAV5 (~10^13^ GC/mL) were injected (2 µL per mouse) bilaterally into the LV (coordinates from bregma: x, −0.58 mm; y, ±1.28 mm; z, −2 mm) with a 10 µL Hamilton syringe (0.2 µL/min). Virus-injected mice were housed for 3 weeks after surgery. Animals were an equal mix of males and females. The *Otx2^+/GFP^* mice, littermates, and the injected *Otx2^lox/lox^* mice underwent transcardial perfusion with 20 mL saline phosphate buffer, and ChPs were dissected and processed for biochemical analysis. Virus-injected mice were sacrificed by cervical elongation for ChP extraction.

### 3.3. Quantitative PCR Analysis

Total RNA from LV and 4V ChPs was extracted by using the RNeasy lipid tissue mini kit (Qiagen, Courtaboeuf, France) with DNA removal. Total RNA (10 to 20 ng) was retrotranscribed by using the QuantiTect reverse transcription kit (Qiagen, Courtaboeuf, France). Quantitative PCR (qPCR) analyses of cDNA (diluted at 1/10) were performed in triplicate with a LightCycler 480 II (Roche, Meylan, France) using the SYBR Green I master mix (Roche, Meylan, France). Gene-to-*Hprt* or gene-to-*Gapdh* ratios were determined by the 2^−ΔΔCt^ method. For *Otx2* expression analysis, expression was compared to mean expression of vehicle-injected mice of the same experiment.

### 3.4. RNA Sequencing Analysis

For analysis of conditional knockdown mice, the RNA was extracted separately from LV and 4V ChPs of Cre-Tat and vehicle-injected mice. A small sample of each ChP was tested by qPCR to ensure Cre-Tat samples had less than 50% *Otx2* expression on average compared to control mice. Duplicate samples were prepared by pooling ChP lysates from 2 × 5 Cre-Tat-injected mice and from 2 × 4 vehicle-injected mice. For analysis of constitutive knockout mice, the RNA was extracted from pooled 4V ChPs of four 3-month-old *Otx2^+/GFP^* and five wildtype littermates, with duplicate samples (*n* = 2) of each genotype. PolyA+ mRNA purification, mRNA sequencing with technical replicates, and data normalization and quantification was performed by the Genomic Paris Center (IBENS, Paris, France) using Illumina HiSeq 1500 (Illumina, Evry, France). 

### 3.5. Isoform Analysis

Raw reads were processed with FASTP [53] using standard parameters, and then pseudo-aligned on mm10 GENCODE transcriptome using salmon [54]. The quantified transcriptome was then imported in R using the IsoformSwitchAnalyzeR package [55,56] with dIFcutoff = 0.15. Isoform switch test was performed using DEXseq [57,58] in IsoformSwitchAnalyzeR. Gene coding potential, secondary structures, signal peptides, and protein domains were analyzed with CPAT [59], Net-Surf2 [60], SignalP [61], and Pfam [62], respectively.

### 3.6. Protein Co-Immunoprecipitation

For each ChP co-IP experiment, ChP from LV and 4V were pooled from four 3-month-old mice and were lysed with 1 mL lysis buffer (100 mM Tris pH 7.5, 1 mM EDTA, 100 mM NaCl, 1% NP40, 1 mM MgCl_2_, 1X protease/phosphatase inhibitor (Roche, Meylan, France)) containing 1µL of benzonase (Roche, Meylan, France). ChP were dissociated using 26G syringe and incubated 30 min on ice. Tubes were centrifuged at 21,000× *g* for 10 min and supernatant was recovered and divided in two. Each half was incubated with 44 µg of either anti-OTX2 (ab21990, Abcam, Paris, France) or anti-IgG (ab27478, Abcam, Paris, France) antibodies coupled to magnetic beads (10 mg/mL with 9.5 µg of antibody per mg of beads, Dynabeads antibody coupling kit, Invitrogen, Vilnius, Lithuania) in lysis buffer at 4 °C on rotating wheel overnight. Using magnetic separation, beads were washed 5 times in 1 mL of cold lysis buffer. Pelleted beads were eluted in 20 µL of laemmli buffer by heating 5 min at 95 °C, and then stored at −20 °C.

For SVZ, RMS, and VCx, tissue was pooled from ten 3-month-old wildtype mice and were lysed by trituration (pipette and 26G syringe) in 10 µL lysis buffer II (20 mM Tris pH 8, 120 mM NaCl, 1% NP40, 1 mM MgCl2, 5% glycerol, 1X protease/phosphatase inhibitor) per mg of tissue supplemented with 1 µL of benzonase per 1mL of lysis buffer II. Samples were processed as described above with 25 µL of antibody-coupled beads. Pelleted beads were eluted in 30 µL of laemmli buffer.

### 3.7. Mass Spectrometry Analysis

Proteomics analyses were performed by the Protein Mass Spectrometry Laboratory (Institut Curie, Paris, France). Eluted samples in laemmli were processed and resulting peptides were analyzed by nano-LC-MS/MS using an Ultimate 3000 system (Dionex, Thermo Fisher Scientific, Paris, France) coupled to an Orbitrap Fusion mass spectrometer (Q-OT-qIT, Thermo Fisher Scientific, Paris, France). Data were acquired using Xcalibur software and the resulting Mascot files (v2.5.1) were further processed by using myProMS software (v3.9) [63]. Percolator [64] was used for FDR calculations set to 1% peptide level. For ChP proteomics, three experiments were performed (*N* = 3). For SVZ, RMS and VCx proteomics, one experiment was performed (*N* = 1). 

### 3.8. Ontology Analysis

Genes with >10 mean reads in at least one of the ChP samples were selected for ontology analysis. Differentially expressed gene lists were generated by using threshold of *p-adj* < 0.05. Ontology term enrichment and KEGG pathways were analyzed with DAVID Bioinformatic resource v6.7 [65,66] and ontology terms were plotted as -log_10_ scale of the enrichment *p*-values. UniProt (access date 21 August 2020, http://www.uniprot.org) was used for obtaining functional classes (Table 1, Table 3, Table 6 and Table 7). Gene list comparisons and Venn diagram data were generated with web-based tools (http://www.bioinformatics.lu/venn.php).

## 4. Conclusions

The ChP has barrier functions for controlling what gets in and out of the brain, and homeostasis functions for controlling brain metabolites in the CSF. The data in this present study allow us to go a step further, by imparting important ChP endocrine functions that are putatively regulated by the cell-autonomous and non-cell-autonomous activities of OTX2. Indeed, in addition to regulating the expression and post-transcriptional modification of genes encoding signaling and hormone-transport proteins that are secreted into the CSF, such as *Igf2* and *Ttr*, OTX2 itself is secreted by the ChP and exerts essential non-cell-autonomous activities, such as the regulation of cerebral cortex plasticity or that of adult neurogenesis [5,19,67]. Transcriptomic analysis of different genetic *Otx2* loss-of-function models, including conditional knockdown, specifically in the ChP, coupled with proteomic analysis, are the first steps towards a better understanding of the molecular biology of this traveling transcription factor in and out of its main cerebral source.

## Figures and Tables

**Table 1:** Top 50 genes expressed in lateral ventricle (LV) and fourth ventricle (4V) choroid plexus (ChP). Significantly different expression between structures is indicated in bold.

Gene Symbol	Function	Mean Reads Combined	Mean Reads 4V ChP	Mean Reads LV ChP	Fold Change LV vs. 4V	*p-adj*
*Ttr*	T_4_ and retinol transport	2,623,541	2,348,304	2,898,779	1.2	0.4700
*Enpp2*	Extracellular signaling	593,932	612,769	575,094	0.94	1
*Malat1*	RNA processing	112,726	157,395	68,058	0.43	0.1482
*Apoe*	Lipid transport	64,482	54,600	74,365	1.4	0.7231
*Trpm3*	Cation channel	39,627	46,150	33,105	0.72	0.1606
*Bsg*	Cell adhesion	49,354	41,701	57,007	1.4	0.5023
*Kl*	Cell signaling	45,044	40,667	49,422	1.2	0.4792
*Abhd2*	Lipid metabolism	41,094	36,405	45,783	1.3	0.2413
*AY036118*	Hemopoiesis	25,992	33,903	18,081	0.53	1
*Slc4a10*	Solute transport	30,242	30,563	29,920	0.98	1
*Psap*	Trophic, metabolism	33,695	28,768	38,623	1.3	0.0646
*Igfbp2*	IGF-binding	25,336	28,556	22,117	0.77	0.4457
*Hspa5*	ER chaperone	25,426	24,960	25,891	1.0	1
*F5*	Hemostasis	25,758	24,672	26,843	1.1	0.9813
*Slc12a2*	Solute transport	23,869	24,279	23,459	0.97	1
***Ctsd***	APP processing	28,200	23,672	**32,728**	**1.4**	0.0423
*Prlr*	Hormone receptor	27,452	22,728	32,176	1.4	0.7565
*Atp1a1*	Ion transport	22,896	22,067	23,724	1.1	1
***Clu***	Extracellular chaperone	26,747	21,750	**31,744**	**1.5**	0.0323
*App*	Cell adhesion, signaling	19,290	21,197	17,383	0.82	0.4130
***Cntn1***	Cell adhesion	25,888	21,017	30,759	1.5	0.0175
*Atp2b3*	Ion transport	20,580	19,745	21,415	1.1	1
*Ahcyl2*	Solute transport	20,726	19,427	22,025	1.1	0.8161
***Igf2***	Growth hormone	24,978	18,973	**30,984**	**1.6**	0.0123
*Hsp90b1*	Chaperone	18,816	18,514	19,117	1.0	1
*Sptbn1*	Cytoskeleton	18,883	18,276	19,490	1.1	1
*Cpe*	Prohormone processing	17,182	17,449	16,916	0.97	1
*Car12*	Metabolism	18,529	17,277	19,782	1.1	0.7979
*Clic6*	Ion channel	16,953	16,432	17,473	1.1	1
*Strip2*	Cytoskeleton	14,776	16,187	13,366	0.83	0.4745
***Timp3***	Collagenase inhibitor	18,530	15,562	21,499	1.4	0.0292
*Itpr1*	ER Ca^2+^ release	16,252	15,546	16,957	1.1	0.9741
*Kcne2*	Potassium channel	13,861	15,416	12,305	0.8	0.8581
*Cgnl1*	Cell adhesion	15,183	15,292	15,073	0.99	1
***Gpm6a***	Membrane structure	22,456	15,181	**29,732**	**2.0**	0.0000
*Slc4a2*	Solute carrier	15,501	14,912	16,090	1.1	1
*Atp5a1*	Metabolism	15,922	14,766	17,078	1.2	0.7456
*Nsg2*	Vesicle trafficking	15,587	14,669	16,506	1.1	0.8949
*Zbtb20*	Transcription factor	12,600	14,603	10,597	0.73	0.5636
*Stk39*	Stress response	14,417	14,507	14,326	0.99	1
*Tmem72*		14,821	14,343	15,298	1.1	1
*Cab39l*	Cell polarity	15,438	14,311	16,565	1.2	0.8214
*Nedd4*	Ubiquitination	15,837	14,272	17,402	1.2	0.4738
*Macf1*	Cytoskeleton	12,704	14,027	11,382	0.81	0.5999
*Vat1l*		14,589	13,983	15,196	1.1	1
*Hsp90ab1*	Chaperone	14,368	13,756	14,979	1.1	1
*Calr*	Chaperone	13,889	13,606	14,171	1.0	1
*Htr2c*	Serotonin receptor	13,453	13,343	13,564	1.0	1
*Slc5a3*	Solute transport	11,648	13,281	10,015	0.75	0.0914
*Sptan1*	Cytoskeleton, Secretion	11,913	13,161	10,665	0.81	0.3292

**Table 2:** Top 10 differentially expressed genes in choroid plexus of Cre^+^*Otx2^lox/lox^* mice. Genes in fourth ventricle with * are also deregulated upon embryonic *Otx2* knockdown in the hindbrain choroid plexus [2].

Gene Symbol	Mean Reads Vehicle	Mean Reads Cre-Tat	Fold Change	*p-adj*
Upregulated in lateral ventricle ChP				
*Igkv1-135*	0.6	33	61	0.0000
*Slc1a6*	2.1	82	39	0.0000
*Mup5*	123	4119	34	0.0000
*Gpx3*	295	7229	25	0.0000
*Ighv1-67*	1.6	36	22	0.0000
*Saa3*	2.2	47	22	0.0000
*Tnfrsf11b*	6.3	116	18	0.0000
*Cacnb3*	24	325	13	0.0000
*Ndnf*	89	1098	12	0.0000
*Gm4841*	3.8	44	12	0.0000
Downregulated in lateral ventricle ChP				
*Ngfr*	444	83	−5.3	0.0000
*Nrn1*	1924	496	−3.9	0.0000
*B3galt2*	109	31	−3.6	0.0007
*Dazl*	207	60	−3.4	0.0000
*Itga10*	184	54	−3.4	0.0000
*Slc26a7*	1670	533	−3.1	0.0000
*Steap1*	2224	735	−3.0	0.0000
*Defb11*	473	169	−2.8	0.0000
*Entpd3*	240	87	−2.8	0.0001
*Ccl9*	2037	774	−2.6	0.0000
Upregulated in 4th ventricle ChP				
*9030619P08Rik*	0.00	28.6	infinite	0.0000
*Tmigd1*	0.00	33.1	infinite	0.0000
*A730020M07Rik*	1.3	76	61	0.0000
*Gpx3*	181	10,780	59	0.0000
*Cacnb3*	18	778	43	0.0000
*Mup5*	26	1053	41	0.0000
Fmod *	286	8688	30	0.0000
*Slitrk6*	4.7	125	27	0.0000
*Ndnf*	84	2070	25	0.0000
*Adcy8*	10	233	23	0.0000
Downregulated in 4th ventricle ChP				
*Ngfr*	684	159	−4.3	0.0000
Steap1 *	1225	326	−3.8	0.0000
*Elfn1*	104	28	−3.7	0.0000
*Gnmt*	122	33	−3.7	0.0000
*Gm22650*	141	40	−3.5	0.0000
*Mir448*	95	28	−3.4	0.0002
*Igf2os*	89	26	−3.4	0.0005
*B3galt2*	141	44	−3.2	0.0000
*Slc26a7*	843	271	−3.1	0.0000
*Crhr2*	504	164	−3.1	0.0000

**Table 3:** Choroid plexus genes with significant expression changes in both *Otx2* knockdown models, including lateral ventricle and fourth ventricle from Cre^+^*Otx2^lox/lox^* mice and fourth ventricle from *Otx2^+/GFP^* mice. Genes with * are also deregulated upon embryonic *Otx2* knockdown in the hindbrain choroid plexus [2].

Up-Regulated	Function	Downregulated	Function
*Adora1*	Adenosine receptor	Aqp1 *	Osmotic gradient
*Arrb1*	Receptor signaling	*Atp2b4*	Ion transport
Atp1a2 *	Ion transport	*B3galt2*	Glycosylation
*Cadm1*	Cell adhesion	*Elfn1*	Signaling cascade
*Cd55*	Complement cascade	*Entpd3*	
*Cfap46*		*Fam132a*	Glucose uptake
*Chn2*	Signaling cascade	*Igf2*	Growth factor
*Col11a1*	Collagen II fibrils	*Ins2*	Glucose uptake
*Col1a2*	Collagen I fibrils	*Kalrn*	Signaling cascade
*Edn3*	Vasoconstriction	*Klhl36*	Ubiquitination
*Eva1a*	Cell death	*Mapk9*	Cell signaling
*Fam211b*		Myo5b *	Cell trafficking
*Fgf1*	Growth factor	Myrip *	Cell trafficking
*Flrt1*	FGF signaling	*Nav3*	Immune response
Fmod *	Collagen I and II fibrils	*Otx2*	Transcription factor
*Gda*	Metabolism	Pcnx *	
*Gpx3*	Oxidative stress	Pitpnm1 *	Cytoskeleton
Hopx *	Chromatin structure	*Pomgnt1*	Metabolism
*Layn*	Hyaluronan receptor	*Rcn1*	ER regulation
*Lrrc18*	Spermatogenesis	*Scg5*	Cell secretion
*Mapk10*	Cell signaling	*Sfrp1*	Wnt signaling
*Matn2*	Extracellular matrix	*Slc29a4*	Cation transport
Megf11 *	Cell adhesion	Slc2a12 *	Glucose transport
*Mlc1*	Osmotic gradient	*Slc35f1*	Solute transport
*Mup5*	Pheromone activity	*Slc41a2*	Magnesium transport
*Ndnf*	Cell adhesion, growth	*Stra6*	Retinol transport
*Ndrg3*		*Tbc1d2*	Cell adhesion
*Pi15*	Protease inhibitor	*Tbcd*	Cytoskeleton
*Plin4*	Adipocyte formation	*Thumpd3*	
*Rufy4*	Autophagy	*Tmem255b*	
*Sel1l3*		*Tmprss11a*	Cellular senescence
Sema5a *	Cell adhesion	Tspan33 *	Notch signaling
*Shisal1*		*Ttr*	Retinol and T_4_ transport
*Smrp1*	Cilia function	Wdr17 *	
Sncg *	Neurofilament network		
Sned1 *			
*Sorcs2*	Signaling cascade		
*Sorl1*	Cell trafficking		
*Sulf2*	Extracellular matrix		
*Tm4sf1*			
*Vim*	Cell filaments		
*Vwa5b1*			

**Table 4:** Expression of secreted factors in *Otx2* knockdown experiments.

Gene	Choroid Plexus	Mean Reads, Control (Either Veh or Wildtype)	Mean Reads, Knockdown (Either Cre-Tat or *Otx2^+/GFP^*)	Fold Change	*p-adj*
*Bmp7*	*Otx2^lox/lo^*^x^ LV	5125	7090	1.4	0.1845
	*Otx2^lox/lox^* 4V	2547	4036	1.6	0.0014
	*Otx2^+/GFP^* 4V	2215	2261	1.0	1
*Wnt2b*	*Otx2^lox/lo^*^x^ LV	10	66	6.6	0.0002
	*Otx2^lox/lox^* 4V	12	66	5.4	0.0001
	*Otx2^+/GFP^* 4V	29	35	1.2	1
*Tgm2*	*Otx2^lox/lo^*^x^ LV	276	422	1.5	0.2496
	*Otx2^lox/lox^* 4V	138	265	1.9	0.0040
	*Otx2^+/GFP^* 4V	388	404	1.0	1
*Sfrp1*	*Otx2^lox/lo^*^x^ LV	8536	4511	0.53	0.0000
	*Otx2^lox/lox^* 4V	5121	2726	0.53	0.0000
	*Otx2^+/GFP^* 4V	4087	2001	0.49	0.0392
*Sostdc1*	*Otx2^lox/lo^*^x^ LV	8627	4429	0.51	0.0000
	*Otx2^lox/lox^* 4V	5412	2793	0.52	0.0076
	*Otx2^+/GFP^* 4V	8932	8458	1.1	1
*Shh*	*Otx2^lox/lo^*^x^ LV	0	1.4	infinite	1
	*Otx2^lox/lox^* 4V	0.7	4.6	6.6	1
	*Otx2^+/GFP^* 4V	0	64	infinite	0.0000
*Slit2*	*Otx2^lox/lo^*^x^ LV	2302	2674	1.2	1
	*Otx2^lox/lox^* 4V	3471	4803	1.4	0.7159
	*Otx2^+/GFP^* 4V	4323	6099	1.4	0.6487
*Fgf2*	*Otx2^lox/lo^*^x^ LV	140	123	0.88	1
	*Otx2^lox/lox^* 4V	64	84	1.3	0.9878
	*Otx2^+/GFP^* 4V	67	66	1.0	1
*Areg*	*Otx2^lox/lo^*^x^ LV	0	0.9	infinite	1
	*Otx2^lox/lox^* 4V	NA	NA	NA	NA
	*Otx2^+/GFP^* 4V	0	0	NA	NA
*Tgf-α*	*Otx2^lox/lo^*^x^ LV	2140	1425	0.67	0.0620
	*Otx2^lox/lox^* 4V	1357	999	0.74	0.1611
	*Otx2^+/GFP^* 4V	1040	1673	1.6	0.2163
*Tgf-β2*	*Otx2^lox/lo^*^x^ LV	9233	4747	0.51	0.0000
	*Otx2^lox/lox^* 4V	3755	2169	0.58	0.0000
	*Otx2^+/GFP^* 4V	3324	2563	0.77	0.3602
*Igf2*	*Otx2^lox/lo^*^x^ LV	42,336	19,144	0.45	0.0008
	*Otx2^lox/lox^* 4V	18,717	8087	0.43	0.0000
	*Otx2^+/GFP^* 4V	42,542	17,197	0.40	0.0000
*Igfbp2*	*Otx2^lox/lo^*^x^ LV	30,195	20,992	0.70	0.3255
	*Otx2^lox/lox^* 4V	28,166	13,235	0.47	0.0003
	*Otx2^+/GFP^* 4V	47,529	21,660	0.46	0.0000

**Table 5:** Summary of protein lists and criteria for identification of putative OTX2 protein partners.

List Name	Choroid Plexus	SVZ	RMS	Visual Cortex
Total proteins OTX2	4814	1138	2425	2644
Total proteins IgG	3602	1776	2274	2667
Unique proteins OTX2 (≥3 peptides)	392	17	40	29
Unique proteins IgG (≥3 peptides)	59	139	22	25
Selected proteins OTX2 (≥50% rel. ∆)	653	6	75	37
Unique small proteins Otx2 (≤25 kDa)	182	31	68	48
Total OTX2 partners	1195 of 4814	52 of 1138	180 of 2425	109 of 2644

**Table 6:** High-confidence OTX2 putative protein partners in choroid plexus identified by MS analysis. Proteins indicated in bold are unique for OTX2 co-IP, proteins with * are “tier 1” stress granule proteins.

Protein	Function	Protein	Function
ABCF1 *	Translation	MAP4	Cytoskeleton
ACOT11	Lipid metabolism	MCM3AP	RNA export
AGO1 *	RNA silencing	MECP2	Transcription, epigenetics
APC	Cell adhesion	MLYCD	Metabolism
**ARHGEF6**	Trafficking	MOV10 *	RNA and LINE-1 silencing
ARHGEF7	Trafficking, cell adhesion	MSI2 *	Translation
ARVCF	Cell adhesion	MYCBP2	Transcription
CDH2	Cell adhesion	**NFATC2**	Signaling
CDH3	Cell adhesion	PIKFYVE	Trafficking
CHD4	Cell adhesion	PITPNM2	Trafficking
CTNNA1	Cell adhesion	POLDIP3	Translation
CTNNA2	Cell adhesion	PRRC2A *	RNA splicing, stress granule
CTNNB1	Cell adhesion	RBM39	RNA splicing
DDX41	RNA splicing	RHOT1	Mitochondrial trafficking
EDC4 *	RNA processing	RPL19	
EPB41L5	Cell adhesion	RPL21	Translation
ERBIN	Signaling	RPL22	
**FIG4**	Trafficking	RPL29	Translation
FMNL3	Cytoskeleton	RPL35	Translation
GIT1	Trafficking, cell adhesion	RPL36A	
**GIT2**	Trafficking	SRRM2	RNA splicing
GJA1	Gap junction	STRAP *	Stress response
GPAM	Metabolism	TJP2	Cell adhesion
GTPBP1	RNA processing	TMPO	Nuclear membrane
HNRPLL *	RNA splicing	TNS2	Signaling
ILF2	Transcription	TRPV4	Osmotic sensitivity
KIFAP3	Chromosome structure	**VAC14**	Trafficking
LBR	Metabolism	VRK3	Signaling
LIG3	DNA repair	**WDR70**	
MAP1A	Cytoskeleton	ZFR	RNA export

**Table 7:** Peptide number comparisons of OTX2 putative protein partners common to non-cell-autonomous structures. Cell-autonomous partners are indicated in bold.

Protein	Function	SVZ		RMS		VCx		ChP	
		Otx2 co-IP	IgG co-IP	Otx2 co-IP	IgG co-IP	Otx2 co-IP	IgG co-IP	Otx2 co-IP	IgG co-IP
ACIN1	mRNA splicing	3	0	11	1	16	4		
**ACOT11**	Lipid metabolism	4	0	5	0	8	1	**34**	**7**
ARCN1	Protein transport	3	1	7	2	4	1		
DDX46	mRNA splicing	30	0	45	1	34	3		
EIF4A3	mRNA translation	4	0	10	2	11	5		
**FIG4**	PI(3,5)P2 regulation, MVB	20	0	26	0	27	0	**43**	**0**
**KCND3**	Potassium channel	6	0	5	0	7	0	**6**	**0**
**PIKFYVE**	PI(3,5)P2 regulation, MVB	36	0	42	0	59	0	**122**	**1**
RBM25	mRNA splicing	5	0	11	1	12	3		
SF3A1	mRNA splicing	3	0	11	3	13	6		
SF3B1	mRNA splicing	3	0	22	0	25	9		
SNRNP200	mRNA splicing	5	0	14	1	31	4		
THOC2	mRNA export	3	0	5	0	3	0		
**VAC14**	PI(3,5)P2 regulation, MVB	39	2	49	3	56	0	**75**	**0**

**Table 8:** List of KEGG pathways associated with putative cell- and non-cell-autonomous OTX2 protein partners.

KEGG Pathway	Size	*p-adj*
Choroid plexus (1326 proteins)		
Tight junction	27 of 137	0.0000
Metabolic pathways	77 of 1184	0.0000
Protein processing in ER	26 of 169	0.0000
RNA transport	24 of 168	0.0000
Ribosome biogenesis	18 or 86	0.0000
Regulation of actin cytoskeleton	27 of 216	0.0000
Ribosome	19 of 119	0.0000
Spliceosome	20 of 138	0.0000
Oxidative phosphorylation	20 of 147	0.0000
Axon guidance	18 of 131	0.0000
SVZ (79 proteins)		
Ribosome	15 of 119	0.0000
Spliceosome	15 of 138	0.0000
RNA transport	4 of 168	0.0004
mRNA surveillance	2 of 93	0.0219
Neurotrophin signaling	2 of 131	0.0297
RMS (219 proteins)		
Spliceosome	28 of 138	0.0000
Oxidative phosphorylation	11 of 147	0.0000
Metabolic pathways	24 of 1184	0.0000
Alzheimer disease	10 of 188	0.0000
mRNA surveillance	8 of 93	0.0000
Visual cortex (192 proteins)		
Spliceosome	17 of 138	0.0000
RNA transport	7 of 168	0.0000
Metabolic pathways	14 of 1184	0.0005
Insulin signaling	5 of 137	0.0007
Oxidative phosphorylation	5 of 147	0.0007

## Data Availability

RNA sequencing data are available with GEO accession GSE157386. Mass spectrometry data are available through the ProteomeXchange Consortium via the PRIDE repository with identifier PXD021244.
